# Adolescents from low-income sectors: the challenge of studying in a time of digital environments

**DOI:** 10.1080/02673843.2014.942792

**Published:** 2014-08-26

**Authors:** Joaquín Linne

**Affiliations:** ^a^ Gino Germani Institute of Investigations, University of Buenos Aires, Buenos Aires, Argentina

**Keywords:** adolescents, low-income sectors, ICTs, study

## Abstract

This paper is about practices and perceptions regarding the study of adolescents from low-income sectors in the City of Buenos Aires. The methodology consisted of 26 in-depth interviews with low-income adolescents and participant observations in twenty cybercafés of the South Area of the City of Buenos Aires. Among the findings, these students highlight that ICTs allow them to handle information in a more agile and entertaining way, more consistent with their daily uses. However, doing research on school content is what students do the least, since adolescents use technology mainly for communicative, social and recreational ends. These adolescents recognise some disadvantages in using ICTs to study: the unreliable information, the difficulty to distinguish which topics related to school content are more appropriate and the disruptive and continuous use of social networks. In this sense, these adolescents tend to have more problems in benefitting from ICTs for academic purposes than other adolescents. While communication and recreational skills tend to be similar, the evaluation of different sources of information and the skill to make complex searches online are usually more strongly developed in adolescents of middle and high-income households. In conclusion, we think it is necessary to take these problems into consideration in the social sciences research of the area and besides when implementing digital literacy programs.

## 1. Introduction

In the last few years, millions of people have started using digital environments with Internet connection. The increase in information and communication technologies (ICTs) has generated new ways of communication, entertainment and processing of information in most cultural areas, specifically among young people (Boyd, 2008; Hinojosa Córdova, 2012; Urresti, 2008).

Today's youth are undergoing a process of socialization that is significantly different from that of older generations. Youth between the ages of eight and eighteen consume an average of seven and one-half hours of digital media per day. Furthermore, the time youth spend online weekly nearly equates to the time an adult would spend at a full-time job. A major portion of that time is spent on social networking platforms. (Cooke Jackson & Barnes, 2013)

The precedent information is specifically about North American adolescents, but also shows global trends in the use of technology among the youth. In this context of adolescents living an important part of their daily lives in digital environments, our mixed research is about the practices and perceptions of adolescent students in low-income sectors in the city of Buenos Aires. When we talk about school practices, we refer to those activities which aim to solve school tasks, and when we talk about ICTs, we refer to technologies in the field of information technologies, microelectronics and telecommunications (Castells, 2009).

In this context, low-income adolescents in Buenos Aires face two problems regarding the use of ICTs at school: on the one hand, socio-economic differences for accessing equipment as well as Internet connectivity and on the other hand the different specific uses of ICTs. Both aspects are connected to the digital gap (Van Dijk & Van Deursen, 2010).

One of the purposes of this research is to show that the pioneering concepts used to describe adolescents have begun to lose meaning. It is necessary, therefore, to conduct a more complex analysis from a sociological perspective regarding the ways society makes use of ICTs. With this intention, our work focuses on the academic uses of Internet in adolescents from low-income sectors. We have noted that the uses of the Internet in this part of the population have specific modalities where the concepts of ‘digital natives’ (Prensky, 2001), ‘born digital’ (Palfrey & Gasser, 2008), ‘post-alpha generation’ (Berardi, 2009), ‘multimedia generation’ (Morduchowicz, 2010) and ‘internet natives’ (Gui & Argentin, 2011) cannot describe them so clearly.

We have also decided to do some research on low-income sectors' adolescents because they face more difficulties; the economic, social, educational and housing difficulties are often shared with other problems, such as social stigma, gender violence, addictions and early pregnancy. Apart from the difficulties they typically face, their daily lives include various activities: going to school; working in poor labour conditions (with irregular schedules, not protected by laws); taking care of the children of the family and, in some cases, working and participating in arts, sports, crafts and computer workshops. In their free time, they get together with friends; chat on Whatsapp and send text messages over the cell phone; spend time with the ‘Play’^1^; watch audio-visual content online; display, take and edit photos and use social network sites. In this context, Facebook appears as their main entertainment and communication media.

## 2. Methodology

This study used a qualitative research approach to explore the phenomena experienced by adolescents from low-income sectors and their use of ICTs as students. The aim of the qualitative research is to gain an understanding of the ways in which individuals or groups experience certain phenomena and the different types of meaning built from their experiences (Symon & Cassell, 2004). The descriptions obtained from adolescents regarding this problem can be analysed in order to provide us with an understanding of their experiences around the investigation's topic (Patton, 2002). Qualitative research also enables several methods of data collection which, at the same time, helps us obtain participants' points of view (Patton, 2002). In a major part of qualitative research, multiple sources of data – interviews, observations, content analysis – are used in order to address a comprehensive understanding of the research problems.

### 2.1. Data collection

This 2-year study was performed through the years 2012 and 2013. The interviews were performed in five schools and four community centres in the south of the city with a majority of adolescents from the low-income sector population. Also, participant observations were performed in 20 cybercafés of the southern area of Buenos Aires City.

By means of the ‘snowball method’ (Symon & Cassell, 2004), we conducted 26 in-depth interviews with low-income adolescents, 14 girls and 12 boys between 12 and 18 years old; their average age was 15 years. The open-ended individual interviews, recorded with the written consent of the participants, were performed in a conversational and casual style, asking about student and technological issues. Among other aspects, questions were related to their daily use of Internet, their main practices around social networking, digital devices owned or used by them, their searches for information using browsers, their computer classes at school and their ‘digital skills’ (Litt, 2013). These skills' measurements were based on adolescents' responses to questions on their ability to perform specific Internet-related tasks. In the interviews, the adolescents responded to questions about many aspects of their life, but this paper is limited to the categories linked to the educational uses of ICTs. All interviews lasted between 50 and 60 minutes and were conducted at schools or community centres, with the consent of the institutional authorities and the participants of the study. In the last 20 minutes of the interviews, we performed participant observations requesting adolescents to carry out different tasks using a netbook: search for general topics through browsers; collect information in order to accomplish a typical homework; use of social networks.

A significant part of the fieldwork was possible thanks to our activity as volunteer teachers at the ‘Conviven’ community centre, located in Lugano, in the southern area of Buenos Aires. In 2012, we collaborated with the computing workshop and also mentored adolescents in English and Literature, where we made our main participating observations using low-income sector adolescents. Half of the participants of the target sample were recruited mainly in this community centre, and the other half at schools, cybercafés and fast food shops in the southern area of the city.

### 2.2. Data analysis

After transcribing the interviews and the fieldwork notes of multiple observations, we began the data analysis. We read the transcripts several times in search of ‘meaning units’ more than preconceptions (Symon & Cassell, 2004). We labelled the main issues by comparing the meaning units and linked them to their socio-economic contexts. The data gathered were segmented, coded and analysed: the segmentation process involves dividing data into main units; coding refers to the process of attaching labels to units; finally, the analysis involves the meanings that come from the segmentating and coding processes (Symon & Cassell, 2004).

The aim of this qualitative process is to determine what segments of data are more important than others for the research in question. This process of analysis evolved through inductive coding, where themes are established during the data review process (Mertens, 1997). This descriptive phase of data analysis was followed by the interpretive phase, where conclusions were made in order to address the research ﬁndings.

### 2.3. Participants

We have worked with a non-probability sample of an intentional type, consisting of adolescents from low-income sectors. Using some indicators from the National Statistics and Census Institute (INDEC, 2012), we define low-income adolescents as those who are between 12 and 18 years, whose parents have not reached a complete secondary school level, who live in neighbourhoods in the South of Buenos Aires City, with lack of at least one public service (lighting, public cleaning, paving, etc.) or in houses with lack of at least a basic social service (gas network, drinking water services, sewage, etc.). Most of the adolescents of the sample have half-employed or unemployed parents or, in the case of their mothers, who mainly work as maids in households of middle and upper sectors in the North and Central Areas of Buenos Aires City.

For ethical reasons, we worked anonymously with the material collected, meaning that no personal data were saved. Although texts mentioned have the express permission of the interviewees, we used generic labels in every extract of the mentioned interview to preserve their identity. This is why in the interview fragments we only revealed the respondents' gender and their social sector.

## 3. Findings: low-income students studying with the ICTs

The following section addresses the most important issues of the study. At first glance, the adolescents use the Internet mainly in social networks, instant messaging chat and for searching information on Google (Linne, 2013). Given the circumstances, doing research on school content is what students do the least because adolescents use technology mainly for communicative, social and recreational ends (Linne & Basile, 2013).

### 3.1. Advantages

In relation to academics, for most of the students, the Internet represents free access to different reference materials. Moreover, they say their main choice to look for school information is Wikipedia. Apart from appreciating the common recreational-communicational uses, students often highlight the fact that ICTs allow them to handle information in a more agile and entertaining way, more consistent with their daily uses. For example, as well as watching video clips or movies on YouTube, tutorials and educational videos are also useful to students. Also, the site they use the most to research school information is Google.

In the interviews, adolescents from both social sectors state that they use the Internet to look for information related to their studies: ‘You can save more time than when looking up information in books’ (Male, 14 years old). For them, it is easier and more ‘natural’ to look for material in Google than in the school library: ‘We just go to the Internet to do all the homework’ (Female, 15 years old). However, many times they do not have an Internet connection in the institution. Therefore, they do research at home, in cybercafés or in places where they can connect to a public network.

Besides, adolescents think that the Internet allows them to have access to a large variety of different materials: ‘What is good about the Internet is that everything is at hand’ (Female, 17 years old). They also highlight the possibility of finding educational content in audio-visual formats. In many cases, teachers emphasise that students know how to use the Internet, but they cannot differentiate the quality of content. However, adolescents value that ‘the vast amount of information helps you look for important things’ (Female, 15 years old). In this way, for low-income adolescents, looking for information on the Internet is an activity which is easy and agile and which does not imply any other cost other than access to an Internet-enabled computer. In this sense, for adolescents, the use of ICTs not only does not conflict with school requirements, but conversely it makes studying more attractive and tends to simplify the completion of school tasks.

### 3.2. Disadvantages

In the first place, negative considerations include, but are not limited to, unreliable information and the difficulty to distinguish which topics related to school content are more appropriate. The answers given by students to the question ‘What is the negative side of the Internet?’ include the following: ‘A lot of inaccurate information’ (male, 16 years old); ‘It's full of lies and it's difficult to find what is true’ (male, 13 years old); ‘Many times they upload nearly anything, just nonsense, and you waste time trying to find useful information’ (male, 14 years old) and ‘Sometimes it is useful for studying, but sometimes it isn't’ (male, 18 years old). Adolescents emphasise that in the presence of such a lot of information, they find it difficult to identify the right sources to be able to do their school tasks. Also, they pinpoint that through social networking they chat about study, but they do not study.

In addition to all the inaccurate information, they state that continuous diversion is another negative aspect of ICTs, ‘I finish my school tasks as soon as possible or I don't even do them, and I spend my time playing networked games or chatting with my friends’ (male, 16 years old); ‘The Internet distracts you from important things’ (male, 14 years old) and ‘You enter there to see something about your studies and you end up seeing nothing’ (male, 17 years old). In many cases, the distraction becomes a bigger problem when due to it adolescents neglect their family relationships, their peer group and their studies. In this sense, a male (14 years old) says that many adolescents ‘get completely hooked on the computer, especially with games, social networks and porn’. In Peru (Bossio, Bossio, & León, 2012) and other countries as well, adolescents from lower classes use the computer largely to play, chat and use Facebook.

Therefore, studying, which requires a certain degree of concentration and getting into focus, is usually affected by the hyper-connectivity and the hyper-stimulation that the omnipresent online social networks and different games sites, the entertainment and the free-access cultural consumption offer students. In conclusion, Table 1 below identifies the main advantages and disadvantages low-income adolescents find in the use of ICTs regarding their studies.

**Table 1:** Advantages and disadvantages in the use of school ICTs according to low-income adolescents.

Advantages	Disadvantages
Extensive information	Suspicious or inaccurate information
Free-of-charge	Excessive advertising
Easy access	Distraction
Speed	Neglecting face-to-face relationships

Note: Fieldwork-based information.

On the one hand, extensive information is free of charge, easily accessible and fast and appear, among low-income adolescents, as the main virtues they highlight about studying in times of digital environments. On the other hand, suspicious and inaccurate information, continuous interruptions by personalised advertisements and the temptation to get distracted with the use of games, audio-visual content and social networks usually affect their school performance, as well as their face-to-face relationships. Based on the fieldwork, we observed that the different uses of ICTs among adolescents tend to increase the differences in school performance according to the social area.

## 4. The digital and social divides

The concept of ‘digital divide’ (DiMaggio & Hargittai, 2001) appeared in the 1990s together with the concept of ‘Knowledge Society’ (UNESCO, 2005). Although initially it was used to designate those who had or did not have access to information and telecommunication technologies, soon it also became useful to differentiate between those who used this technology for more diverse or more reduced uses.

After over two decades of globalisation of the Internet, the discussion and analysis of the different uses of the ICTs becomes a central issue for public debate which tries to diminish the inequalities among social sectors (Castells, 2009). This is the reason why we will focus on the analysis of the socio-economic and housing restrictions which tend to generate unequal uses of technology.

### 4.1. Restrictions

The main restriction is the fact of having or not a terminal with Internet access. In this regard, those who have a personal computer are clearly favoured compared to those who have to use somebody else's computer, whether outside or inside their homes, or those who have to share the computer with other family members. In all these cases, the availability of the computer is relative and limited, and there is less privacy in the use of it. This phenomenon is typical of low-income sectors.

The place where the computer is used has an influence on school performance. In low-income sectors, we can see this in terms of the lack of space to study or to have an office or their own bedrooms. In this sense, the solution proposed by the one-to-one plans with regards to accessing a personal terminal does not solve the spatial or the connectivity problem. Therefore, low-income adolescents have more difficulties to study at home than middle-class adolescents, who, in most cases, have their own bedroom, a place to study and at least one computer of their own with Internet connection. The development and implementation of virtual classrooms and online social network study groups should not distract us from the importance of having an adequate physical space to be able to fulfil school tasks satisfactorily.

Moreover, low-income students' parents find it very difficult to help their children with their school and computer tasks due to their low educational level and their poor knowledge of ICTs. By contrast, although they are digital immigrants, middle-class adolescents' parents have, in most cases, more computing knowledge associated to a higher educational level than low-income parents. These skills are usually due to the fact that middle-class adults have, more often than low-income parents, professional or administrative jobs which require an intensive use of ICTs (INDEC, 2012). This is another example of how digital inequality is directly affected by the educational inequality (Wei & Hindman, 2011). These factors must be considered when evaluating the students' use of the ICTs according to their social sector, taking into account the limits and obstacles that may arise from the programmes based on the one-to-one model.

### 4.2. One-to-one plans

In the mid-nineties, with the aim of taking advantage of the potentially equalising resources of ICTs in the educational area, Negroponte, one of the main pioneers in generating plans to diminish the digital gap, had the idea of giving a computer to every child and adolescent attending school.

This project attracted the interest of governments in different parts of the world. In Latin America, the main public programmes which have implemented it are the pioneers *Plan Ceibal* (Uruguay), *Proyecto Conectar Igualdad* (Connecting Equity Project) (Argentina), *Un computador por alumno* (A Computer for Each Student) (Brasil), *Una laptop por niño* (A Laptop for Each Child) (Peru), *Proyecto Piloto uno a uno* (One to One Pilot Project) (Colombia), *Canaima Project* (Venezuela), *Modelo pedagógico 1:1* (1:1 Pedagogic Model) (Paraguay), *Cerrando la Brecha del Conocimiento* (Closing the Gap of Knowledge) (El Salvador) and *Proyecto de Tecnologías Móviles* (Mobile Technologies Project) (Costa Rica). While public programmes are implemented to incorporate ICTs to the educational system, and to extend and improve digital literacy between the youth, it is worth considering how adolescents use these tools, what obstacles they face when they use them at school and how social restrictions affect their use.

As mentioned above, public programmes based on the one-to-one model usually have the objective to respond to inequalities in access to computers, to provide digital literacy to disadvantaged students and therefore to diminish the digital gap. Regarding the Argentinean *Proyecto Conectar Igualdad* (Connecting Equity Project), we must take into account that the supply of equipment, teacher training and the necessary logistic for its correct implementation have not finished yet, and they are part of a long process of infrastructure set-up and digital literacy for teachers and non-teaching staff as well as for the students (Linne & Basile, 2013). For example, maintenance and unlocking of computers usually present difficulties due to the lack of staff trained do it. Therefore, just having received netbooks does not guarantee their satisfactory use. In many cases, the slow and bureaucratic process to unlock them results in the students not using them anymore.

They gave me the netbook, I used it for a few months, but one day it broke down; I returned it to have it repaired and they took a very long time to do it. Some months later, they gave it back to me but it got locked and so I stopped using it. (Male, 16 years old)

When my computer got locked for the fourth time, I stopped taking it to school. You waste more time trying to have it repaired, than using it. Then, they are left somewhere at school and there's nobody to fix them. (Female, 15 years old)

Those students who have another computer at home will be able to go on with their school tasks. In this way, once again, low-income students are disadvantaged, and they usually have more difficulties in their media literacy process. In addition, they explain that the Internet they learn at school is not ‘their Internet’ and, in general, it is not useful to develop their interests and address their concerns.

In this sense, adolescents of low-income households tend to have more problems in their critical thinking as media consumers. While communication and recreational skills tend to be similar, the evaluation of different sources of information and the skill to carry out complex searches on the web are usually more strongly developed in adolescents of middle and high-income households.

Besides, in addition to accessibility, there is a connectivity problem. Full implementation of free Internet access through public programmes is a pending matter to date because there are still some problems with the Internet signal (slowness and interruptions). Although the netbook is not mainly used for school purposes, it is important especially for group work and network communication tasks.

## 5. Conclusions

Regarding low-income adolescents' ICT use, communication and entertainment go far beyond school tasks. These recreational-communicative uses are similar to those among middle-class adolescents, with whom they share the use of the same digital platforms: Facebook, YouTube, Google and Wikipedia. Also, middle-class adolescents and low-income adolescents alike usually say that the implementation of ICTs at school is favourable because these technologies provide easy, fast and free access to numerous contents.

Regarding negative aspects, they emphasise the problems ICTs cause in concentrating on school tasks; they highlight the distractions that inaccurate information, advertising, videogames, pornography, instant messaging and social networks generate. But even considering these obstacles, adolescents maintain that they prefer to do their school tasks with computers and the Internet rather than in the previous traditional way. Taking into account that incorporation of ICTs into public school is supported by adolescents, it is likely to benefit motivation and interest in their studies.

Schools using ICTs have several socio-economic and cultural restrictions which have a greater impact on low-income adolescents. This can be noticed in several aspects: (1) the difficulty to have access and own a computer; (2) lack of good connectivity to the Internet at home; (3) lack of a place to study at home; (4) obstacles regarding software and hardware maintenance due to lack of specific previous knowledge at school as well as at home and (5) parents' limitations, due to lack of computer literacy, to control and accompany their children with their school tasks.

Generations born and raised in the nineties with the massification of digital technologies are called ‘digital natives’, and with the unequal proliferation of ICTs among societies, a gap arose within the generation of digital natives. Adolescents of low-income households tend to have less connectivity and digital environments to improve their technological skills than adolescents of middle and high-income households. In this context, several investigations and institutions started generating strategies to fight inequality within the generation of the so-called digital natives. Because not all adolescents have access to computers in the same way, some programmes like *Proyecto Conectar Igualdad* aim to reduce this inequality through the universal distribution of computers to students in secondary public schools. But even when this process comes to an end and all adolescents have a computer with Internet connection, school uses will still tend to be unequal.

Socio-economic differences and differences in computer resources at home and at schools are part of a scenario that impacts the unequal school performance associated with ICTs in many ways. Therefore, it is necessary to incorporate such problems to the analysis of social sciences and to take them into consideration when designing study plans and implementing literacy programmes. If a greater series of practices and technological literacy are incorporated into school curriculums, the digital gap will tend to reduce in its most relevant aspect: educational practices.

Finally, being a non-intentional sample, findings are not extrapolated to the entire population. However, we believe that this investigation is a significant contribution because it explores and describes certain tendencies in the use of ICTs between adolescents of low-income sectors in Buenos Aires. Its value lies in that it shows an approach to school use by adolescents from low-income sectors of an important city of Latin America, with the intention of collaborating in future comparative works both within the region and globally.

## Funding

This work was supported by the Consejo Nacional de Ciencia y Técnica (National Council of Science & Technique, Argentina) through a scholarship of doctoral completion.

## Footnotes

^1.^ Meaning Playstation, a well-known brand of videogames.

## References

[cit0001] (2009). Precarious Rhapsody: Semiocapitalism and the pathologies of post-alpha generation.

[cit0002] (2012). El poder de las TIC en el fortalecimiento de las capacidades: el caso de las organizaciones sociales de base en las áreas rurales de los andes peruanos.

[cit0003] (2008). Why youth love social network sites: The role of networked publics in teenage social life.

[cit0004] (2009). Communication and power.

[cit0005] (2013). Peer-to-peer mentoring among urban youth: The intersection of health communication, media literacy and digital health vignettes. Journal of Digital and Media Literacy.

[cit0006] (2001). Social implications of the Internet. Annual Reviews of Sociology.

[cit0007] (2011). Digital skills of Internet natives: Different forms of digital literacy in a random sample of northern Italian high school students. New Media & Society.

[cit0008] (2012). Educación y consumo cultural: Una aproximación a los públicos universitarios. Ciencia, Docencia y Tecnología.

[cit0009] (2013). Adolescentes y redes sociales. Usos y apropiaciones de Facebook en sectores populares de la Ciudad de Buenos Aires *[Adolescents and social networking. Uses and appropiations of Facebook in low-income sectors of the City of Buenos Aires]*.

[cit0010] (2013). Usos escolares de Internet en adolescentes de sectores populares [School uses of the Internet in adolescents from popular sectors]. Espacio Abierto.

[cit0011] (2013). Measuring users' Internet skills: A review of past assessments and a look toward the future. New Media Society.

[cit0012] (1997). Research methods in education and psychology. Integrating diversity with quantitative and qualitative approaches.

[cit0013] (2010). La generación multimedia. Significados, consumos y prácticas culturales de los jóvenes.

[cit0014] National Statistics and Census Institute (Instituto Nacional de Estadística y Censos) (INDEC) (2012). Encuesta Nacional sobre el acceso y uso de las tecnologías de la información y comunicación (TIC). http://www.indec.mecon.ar.

[cit0015] (2008). Born digital.

[cit0016] (2002). Qualitative research and evaluation methods.

[cit0017] (2001). Digital natives, digital inmigrants. On the Horizon.

[cit0018] (2004). Essential guide to qualitative methods in organizational research.

[cit0019] UNESCO (2005). Hacia las sociedades del conocimiento. http://unesdoc.unesco.org/images/0014/001419/141908s.pdf.

[cit0020] (2008). Ciberculturas juveniles: vida cotidiana, subjetividad y pertenencia entre los jóvenes ante el impacto de las nuevas tecnologías de la comunicación y la información.

[cit0021] (2010). Internet skills and the digital divide. New Media and Society.

[cit0022] (2011). Does the digital divide matter more? Comparing the effects of new media and old media use on the education-based knowledge gap. Mass Communication and Society.

